# Dating the origin of a viral domestication event in parasitoid wasps attacking Diptera

**DOI:** 10.1098/rspb.2024.2135

**Published:** 2025-01-22

**Authors:** Benjamin Guinet, Jonathan Vogel, Nabila Kacem Haddj El Mrabet, Ralph S. Peters, Jan Hrcek, Mattew L. Buffington, Julien Varaldi

**Affiliations:** ^1^ Université Lyon 1, CNRS, Laboratoire de Biométrie et Biologie Evolutive UMR 5558, Villeurbanne F-69622, France; ^2^ Centre for Palaeogenetics, Stockholm, Sweden; ^3^ Department of Bioinformatics and Genetics, Swedish Museum of Natural History, Stockholm, Sweden; ^4^ Leibniz Institute for the Analysis of Biodiversity Change, Museum Koenig Bonn, Adenauerallee 160, Bonn 53113, Germany; ^5^ Laboratoire d’Ecobiologie des Insectes Parasitoïdes, Université Rennes 1, Campus de Beaulieu, Rennes Cedex 35042, France; ^6^ Biology Centre of the Czech Academy of Sciences, Institute of Entomology, Branišovská 1160/31, České Budějovice 370 05, Czech Republic; ^7^ USDA-ARS Systematic Entomology Laboratory, Washington D.C., USA

**Keywords:** endogenous viral elements, filamentovirus, parasitoid wasp, HGT, Cynipoidea, palaeovirology

## Abstract

Over the course of evolution, hymenopteran parasitoids have developed a close relationship with heritable viruses, sometimes integrating viral genes into their chromosomes. For example, in *Drosophila* parasitoids belonging to the *Leptopilina* genus, 13 viral genes from the *Filamentoviridae* family have been domesticated to deliver immunosuppressive factors to host immune cells, thereby protecting parasitoid offspring from the host immune response. The present study aims to comprehensively characterize this domestication event in terms of the viral genes involved, the wasp diversity affected by this event and its chronology. Our genomic analysis of 41 Cynipoidea wasps from six subfamilies revealed 18 viral genes that were endogenized during the early radiation of the Eucoilini/Trichoplastini clade around 75 million years ago. Wasps from this highly diverse clade develop not only from *Drosophila* but also from a variety of Schizophora. This event coincides with the radiation of Schizophora, a highly speciose Diptera clade, suggesting that viral domestication facilitated wasp diversification in response to host diversification. Additionally, in one of the species, at least one viral gene was replaced by another gene derived from a related filamentovirus. This study highlights the impact of viral domestication on the diversification of parasitoid wasps.

## 1. Introduction

Parasitoid wasps exhibit remarkable species richness, constituting a major component of insect biodiversity [1]. Their biology is characterized by a peculiar reproductive strategy, wherein the parasitoid wasp larvae develop in (endoparasitoid) or on (ectoparasitoid) their host, mostly other insects. Because they usually ultimately kill their hosts, they are major players in the regulation of insect communities. It is, therefore, of general interest to identify the factors that have facilitated their diversification in order to understand the dynamics of insect biodiversity.

Throughout evolution, endoparasitoid wasps have maintained a special relationship with viruses. This is evident not only from the abundance and diversity of ‘free-living’ heritable viruses that are injected during oviposition into their hosts [2–7] but also from the abundance of endogenous viral elements found in their genomes. In line with this idea, it was recently found that Hymenoptera with an endoparasitoid lifestyle had a higher propensity to endogenize (i.e. integrate into their chromosomes) and domesticate (i.e. retain by selection) genes acquired from dsDNA viruses, compared with ectoparasitoids or free-living Hymenoptera [8]. Some of these endogenous viral elements (EVEs) have played an essential role in the interaction between these wasps and the immunity of their hosts. In some wasps, entire viral machinery has been domesticated, resulting in the production of ‘virus-like structures’ (VLS) in the reproductive apparatus of females. These VLS enable the delivery of either DNA encoding immunosuppressive factors or immunosuppressive proteins to the host’s immune system [9–13]. When VLS contain DNA, in systems known as polydnaviruses (PDVs), the DNA integrates into the host haemocyte’s DNA, gets expressed [14] and subsequently influences the host’s physiology and behaviour, ultimately favouring the development of wasp offspring [14,15]. Alternatively, when VLS contain proteins in systems known as virus-like particles (VLPs), the viral machinery facilitates the entry of virulence proteins into host immune cells, thereby suppressing the host immune response [16,17]. The domestication of viruses has probably contributed to adaptive radiation observed in some highly speciose clades of endoparasitoid wasps, for instance in the microgastroid complex (Ichneumonoidea: Braconidae) following the domestication of an ancestral nudivirus [18,19]. Viral domestication is, therefore, likely to have had a profound effect on the diversification of parasitoid wasps and, in turn, on the regulation of insect communities over time.

Recently, a case of viral domestication has been put forward in the *Drosophila* parasitoids belonging to the genus *Leptopilina* (Figitidae) [13]. Since the 1980s, it has been recognized that *Leptopilina* females protect their offspring by producing VLPs in their venom glands, but the evolutionary origin of the genes responsible for their production was unknown [16,20,21]. Analysis of their genomes revealed the presence of 13 EVEs that are clustered in the wasp genome, with some level of synteny conservation between species, suggesting a single endogenization event predating the diversification of *Leptopilina* species [13]. However, the wasp diversity affected by this domestication event and its chronology remain unclear. These EVEs are specifically expressed in the venom gland during the early pupal stage of the wasp when VLPs are synthesized. Furthermore, a viral DNA polymerase (LbFVorf58) most likely amplifies some of the EVEs (10/13) resulting in a concordant peak in DNA copy number and transcript levels [13]. The intact open reading frames (ORFs) and the very low d*N/*d*S* values (ratio that compares the rate of nonsynonymous substitutions (dN) to the rate of synonymous substitutions (dS) in a protein-coding sequence) for all 13 EVEs further indicate that these genes are under strong purifying selection since their entry into wasp genomes. Finally, one of these EVEs (LbFVorf85) has been detected as a protein in purified VLPs, providing evidence for the involvement of these EVEs in VLP production. Once formed in the venom gland, VLPs are injected into the host along with the egg, allowing the delivery of proteins to host immune cells, ultimately protecting the developing parasitoid from the host immune response [16,22]. The ancestral virus that provided *Leptopilina* species with these genes belongs to a recently proposed family of dsDNA viruses, i.e. the *Filamentoviridae* [7]. *Filamentoviridae* appear to be specialized on hymenopteran parasitoids, as suggested by the fact that all known filamentoviruses only infect endoparasitoid wasps and that endogenous versions of filamentous genes are preferentially and often found in the genomes of Hymenoptera with an endoparasitoid lifestyle, such as, for instance, *Dolichomitus* sp. (Ichneumonidae) [23] and *Platygaster orseoliae* (Platygastridae) [7,8], while they are extremely rare in the genomes of non-parasitoid hymenopterans [7].

So far, all six species examined within the genus *Leptopilina* tested positive for these filamentovirus EVEs (FV EVEs), while two species, attacking similar hosts, belonging to the related genus *Ganaspis*, were negative [13]. This suggests that the endogenization event occurred within Figitidae : Eucoilinae after the split between *Ganaspis* and *Leptopilina* species (approximately 91.1 Myr, [24]) but before the diversification of *Leptopilina* ( <40 Myr, [24]).

However, because *Ganaspis* is quite distantly related to *Leptopilina*, and because several intermediate clades interleaved between those taxa were not tested, the breadth of the Figitidae diversity concerned by this event is currently unknown. In addition, despite the absence of FV-derived genes in *Ganaspis*, it is still possible that the domestication predated the split between *Ganaspis* and *Leptopilina* and was subsequently lost in *Ganaspis*. Additionally, since only one *Filamentoviridae* genome was available at the time that the *Leptopilina* genomes were screened for viral genes, we can expect the discovery of additional genes, now that more FV have been sequenced [7]. In short, our understanding of this major endogenization event remains incomplete both in terms of the diversity of wasps involved and the viral genes involved.

Figitidae are the most speciose family within the Cynipoidea superfamily and play an important ecological role in a wide range of environments by controlling the populations of their widely distributed and diversified hosts [25]. Most of them are koinobiont endoparasitoids (meaning they allow the host to continue development while the parasitoid larva grows) attacking larvae of Schizophora flies. These flies account for one-third of the fly diversity within Cyclorrhapha, with over 55 000 known species [26] and are encountered in all parts of the world in various habitats encompassing leaf-mines, decaying fruits, dung or carcasses [27]. The known diversity of Figitidae is also remarkable knowing the challenge of morphological identification in this clade [28], with more than 1700 species described and 14 000 species currently estimated worldwide [29,30]. Within the Figitidae family, the Eucoilinae subfamily, to which *Leptopilina* belongs, stands out as the most species rich, with around 1000 described species [31–33].

In this article, we first tested whether the viral domestication documented in *Leptopilina* sp. is restricted to Figitidae attacking *Drosophila* larvae living in decaying organic matter, as *Leptopilina* species do, or is rather shared with other Figitidae with different ecology (i.e. attacking non-*Drosophila* hosts and/or living in different habitats). Second, we asked the question: if a new wasp species turns out to share the same orthologous EVEs, will it also be associated with VLP production?

To address these questions, the presence of FV-like EVEs was first investigated in 26 Figitidae species covering most of the Figitidae diversity using a combination of PCR (Polymerase Chain Reaction) and whole genome sequencing. The results revealed a single ancestral endogenization event in the common ancestor of all Eucoilini + Trichoplastini, including *Drosophila* and non-*Drosophila* parasitoids, that occurred around 76.4 million years ago during the late Cretaceous period, roughly coinciding with the timing of Schizophora host diversification. Furthermore, we show that the most early diverging species of this virus-bearing clade, also produce VLPs. Using the whole genome dataset, we additionally identify new genes (apart from the 13 genes identified by [13]) that derive from the same ancestral endogenization event, thanks to recent advances in the delineation of filamentovirus diversity [7]. A second independent endogenization event involving a close relative of Leptopilina boulardi Filamentous virus (LbFV) was also observed in one species, which likely resulted in the replacement of a gene acquired from the ancestral event. These results support the critical role played by filamentovirus core genes in the production of VLPs in endoparasitoids and, more generally, on their diversification.

## 2. Results

### (a) All Eucoilini + Trichoplastini species contain *Filamentoviridae* genes in their chromosomes

**Figure 1.**
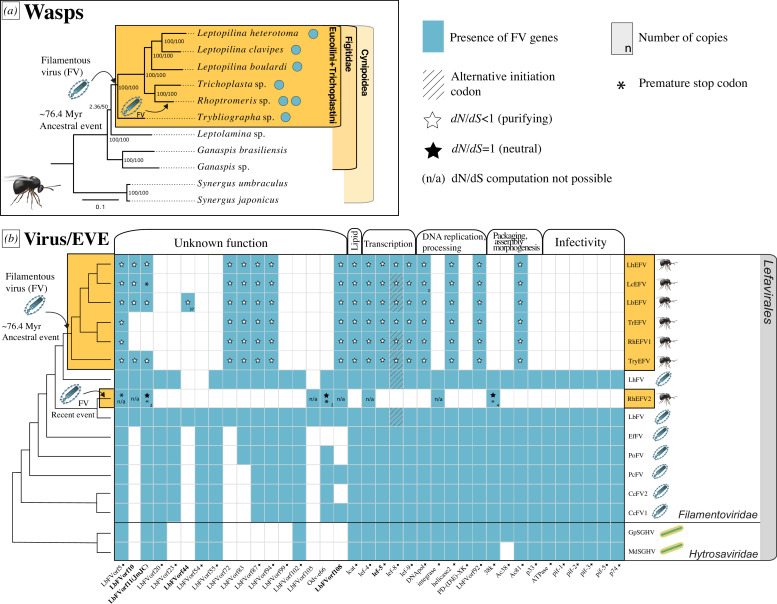
Domestication of *Filamentoviridae* (FV) in Eucoilini + Trichoplastini wasps. (*a*) The phylogeny of the Cynipoidea has been estimated using 1000 BUSCO genes. Confidence scores (approximately Likelihood Ratio Test (aLRT)%/ultra-bootstrap support%) are shown at each node. Blue circles indicate the presence of FV EVE in a genome. (*b*) The heatmap represents the distribution of viral ORF in *Hytrosaviridae* virus and *Filamentoviridae* virus, as well as the distribution of endogenous viral elements (EVEs) from filamentovirus origin in Eucoilini and Trichoplastini wasps. A black diamond symbol is added to the names of viral genes that are part of the core genes of *Naldaviricetes* (= dsDNA arthopod viruses). The cladogram phylogeny of the virus and EVEs is reported on the left and has been made according to the results from electronic supplementary material, figure S6. The rows represent the viral or Eucoilini + Trichoplastini species, and the columns represent the viral ORFs grouped according to their potential functions. A hash indicates that the predicted ORF has an alternative start codon. A white star indicates evidence for purifying selection (d*N/*d*S* < 1) while a black star indicates no evidence for selection (d*N/*d*S* = 1). n/a indicates that d*N/*d*S* computation was not possible because the number of sequences was too small. In case multiple paralogue EVEs were detected, their number is displayed in the bottom right corner. The asterisk corresponds to the presence of premature stop codons in the EVE. When multiple copies are present, a semi-asterisk indicates that some copies have a premature stop codon, while at least one copy has a complete ORF. Gene names highlighted in bold are the EVEs from the ancestral event that are newly described in this study.

In order to investigate the distribution of *Filamentoviridae*-derived genes among Figitidae wasps (and four additional outgroup species belonging to Liopteridae and Cynipidae), a PCR screening was conducted on 41 specimens representing 20 genera divided into 6 subfamilies, i.e. around 145 Myr of wasp evolution [24] (electronic supplementary material, figures S2 and S3). As expected from previous results, the three *Leptopilina* species were positive, while *Ganaspis* was negative. In addition, we found that *Trybliographa* sp. was also positive (electronic supplementary material, figure S2). Following this preliminary PCR screening, we sequenced and assembled the genomes of *Trybliographa* sp., along with two related wasps belonging to the Trichoplastini: *Rhoptromeris* sp. and *Trichoplasta* sp. that were selected because of their position in between *Leptopilina* sp. and *Trybliographa* sp. We also included in our analysis the most basal *Leptolamina* sp. of uncertain tribe [30,34] and the published genome assemblies of *Leptopilina* (for which we utilized the long read assemblies for *L. heterotoma* and *L. boulardi*), as well as the genome assemblies of *Ganaspis* and *Synergus* (Cynipidae: Ceroptresini). The assemblies of the nine species ranged from 354.80 Mb to 935.64 Mb, with a minimum of 93.8% Benchmarking Universal Single-Copy Orthologs (BUSCO) genes (complete and fragmented; see electronic supplementary material, table S1 for all assembly details). Using the predicted proteins from 7 *Filamentoviridae* virus genomes as queries [7], we found significant viral hits in 6 out of 11 genomes: *L. heterotoma*, *L. clavipes*, *L. boulardi*, *Trichoplasta* sp., *Rhoptromeris* sp., *Trybliographa* sp. (Figure 1*a*). On the contrary, no viral hits were detected in the *Synergus* and *Ganaspis* genomes, nor in that of *Leptolamina* sp. In total, for the 6 positive-tested genomes, we identified 153 putative EVEs deriving from the integration of 25 filamentovirus genes (electronic supplementary material, figure S1). Overall, we found 25.6 EVEs/genomes (s.d. = 14.68) with the highest number of EVEs in *L. boulardi* (*n* = 54) and the lowest in *Trichoplasta* (*n* = 15). For detailed filamentous gene alignments, see electronic supplementary material, figure S15. All 107 scaffolds containing the candidate EVEs had coverage and GC (guanine-cytosine) content profiles similar to those of BUSCO scaffolds, providing evidence for their integration within wasp chromosomes (electronic supplementary material, figureS4). In addition, 55 out of the 107 EVE-containing scaffolds, also harboured at least one eukaryotic gene or transposable element, further supporting their chromosomal integration into wasp chromosomes.

### (b) Two independent integrations of filamentovirus occurred in Eucoilini + Trichoplastini

Analysing each of the 25 individual gene phylogenies revealed three topologies that will be briefly described below (electronic supplementary material, figure S5).

In type I phylogenies, all Eucoilini + Trichoplastini sequences formed a monophyletic clade nested within a filamentovirus clade, branching with Leptopilina heterotoma Filamentous virus (LhFV). These phylogenies are consistent with a single endogenization event occurring before the divergence of the six species and involving an LhFV-like donor.

In type II phylogenies, a single sequence from *Rhoptromeris* sp. was nested within a filamentovirus clade, typically branching with Leptopilina boulardi Filamentous virus (LbFV). These phylogenies suggested a single endogenization event specific to *Rhoptromeris* sp. involving an LbFV-like donor.

In type III phylogenies, both type I and type II patterns were observed, indicating an endogenization event involving an LhFV-like donor in the common ancestor of Eucoilini + Trichoplastini species (as in type I) and a second event in the branch leading to *Rhoptromeris* sp. involving an LbFV-like donor (as in type II).

Out of the 25 genes, 5 were left unclassified because of insufficient signal, while 12 exhibited type I, 3 exhibited type II and 5 exhibited type III patterns. All filamentous gene phylogenies can be found in electronic supplementary material, figure S16.

The gene-level phylogenetic analysis thus strongly suggests that two independent endogenization events occurred in this wasp clade: an ancestral event almost basal to the Eucoilini + Trichoplastini and another one more recent and specific to *Rhoptromeris* sp.

**Figure 2.**
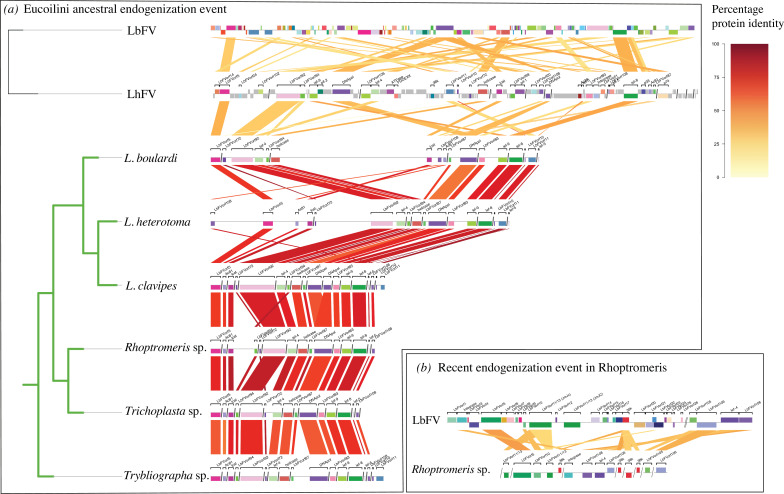
Comparative genomics of wasp scaffolds sharing similarities with filamentous ORFs. (*a*,*b*) The phylogenetic tree on the left has been made according to the results from electronic supplementary material, figure S6. Green and black branches correspond to Eucoilini + Trichoplastini and filamentous branches, respectively. The red/yellow colour code depicts the percentage of protein identity between homologous sequence pairs (viral or virally derived loci). Coloured boxes identify the virally derived genes and their orientation (above: sense; below: antisense). The figure has been drawn using the genoPlotR package [35]. The scaffolds are ordered from left to right in an arbitrary manner. LbFV, Leptopilina boulardi Filamentous virus; LhFV, Leptopilina heterotoma Filamentous virus.

Because EVEs that are physically close to each other (in the same scaffold) are likely to have been acquired during the same endogenization event, we used this criterion to group them. A total of 71 out of the 153 EVEs were found to co-occur on the same scaffolds in one or the other genome assemblies (figure 2). As expected, this genomic-location-based grouping systematically grouped together genes with similar phylogenies supporting the hypothesis that co-location indeed indicates common evolutionary history. This way, it was possible to assign two unresolved gene phylogenies to the two events (LbFVorf44 in the ancestral event and LbFVorf105 in the recent *Rhoptromeris* sp. event). Finally, integrating both phylogenetic and co-location information, we were able to assign 150 of the 153 FV EVEs to the two independent events. In the end, we estimated that 18 and 9 filamentovirus genes have been jointly acquired following, respectively, the first and second endogenization events. Note that the set of filamentovirus genes acquired during these events overlap by five genes (those having ‘type III’ phylogenies). Within each event, the gene phylogenies exhibited congruence, further supporting a shared evolutionary scenario for the different genes assigned to each event. We will, respectively, name these two independent endogenization events: *ancestral event* and *recent event* in the following section.

#### (i) First ancestral endogenization event within the Eucoilini + Trichoplastini common ancestor

The first ancestral event, in the common ancestor of the 6 Eucoilini + Trichoplastini species, involved the endogenization of 18 filamentous genes, including the 13 genes previously identified as domesticated in *Leptopilina* species [13]. Concatenating the viral genes shared by all species (*n* = 15) produced a consistent phylogenetic signal, with Eucoilini + Trichoplastini species forming a highly supported monophyletic clade (bootstrap score = 100) nested within filamentovirus diversity, with LhFV being the closest relative (figure 1*b*). As expected from an ancestral endogenization event, the phylogeny of the EVEs mirrors the evolutionary history of the species (see figure 1*a,b*).

Moreover, the presence of a few conserved gene synteny blocks across Eucoilini + Trichoplastini genomes further supports the conclusion of a single event of endogenization. Notably, gene synteny was particularly evident in the best-assembled genomes of *L. boulardi* and *L. heterotoma*. In both genomes, we observed co-localizations of various genes, such as LbFVorf92/*lef-4*, DNApol/LbFVorf87, LbFVorf83/*lef-9* and LCAT/LbFVorf108/*Ac81* (figure 2*a*). We also noted a gene association between LbFVORF10 and LbFVORF11. This association was not only observed in the free-living viruses LbFV and LhFV but also consistently found in *Leptopilina* species and in the *Trybliographa* genome (figure 2*a*). Altogether these results strongly suggest that the 18 filamentous genes were jointly acquired during a single ancestral endogenization event predating the Eucoilini + Trichoplastini radiation.

Overall, the EVEs identified in the ancestral event were enriched for core viral genes (defined at the level of *Naldaviricetes*) with 12 core genes among the 18 EVEs (compared with a putative donor virus such as LhFV with 110 genes and 29 core genes, Fisher test, odd-ratio = 5.5, *p*‐value = 0.001753). These genes are likely involved in cholesterol metabolism, DNA replication and processing, transcription, packaging and assembly, morphogenesis and unknown functions (figure 1). This enrichment in core genes is in line with the data obtained in other cases of viral domestication in parasitoid wasps [9–12] and suggests that core viral functions are crucial for the production of VLPs.

In comparison with our previous study [13], we identified five additional genes acquired during the same ancestral event (figure 1: highlighted in bold), three of which were shared by all Eucoilini + Trichoplastini species. The gene names, their putative functions and their phylogenies can be found in electronic supplementary material, figure S16 and the alignments in electronic supplementary material, figure S15. These additional genes are briefly described below.

One of these genes, LbFVorf108, is shared by all species from this event (except for *Leptolamina* sp.) and localized next to previously documented EVEs in some of the wasp assemblies (figure 2*a*). The presence of complete ORFs and indications of strong purifying selection (d*N/*d*S* = 0.2792 (s.e. = 0.03)) in all Eucoilini + Trichoplastini EVEs suggests that LbFVorf108 is functional in Eucoilini + Trichoplastini genomes. However, its function is unknown, as no homologues outside *Filamentoviridae* are available in public databases.

The LbFVorf10 and LbFVorf11 (JmJC) were consistently found next to each other on the same wasp scaffolds (figure 2*a,b*). Both genes are also next to each other in the genomes of their closest relatives LhFV and LbFV, which further suggests they entered into wasp genomes together during each of the two independent events. They have been jointly lost in the related *Trichoplasta* sp. and *Rhoptromeris* sp. All LbFVorf10 EVEs presented a complete ORF and a d*N/*d*S* analysis on the EVEs suggested strong purifying selection (mean d*N/*d*S* = 0.1654, s.e. = 0.033). HHpred, a bioinformatics tool that uses hidden Markov models (HMMs) to detect homology by comparing a query protein sequence to databases of known structures and functions, revealed homology between LbFVorf10 and a glycoprotein containing a zinc finger domain from a Hantavirus protein (e-value = 5.9 × 10^−8^) (electronic supplementary material, table S3). Homologues of LbFVorf11 in *L. heterotoma* and *Trybliographa* sp. were under purifying selection but showed either no signs of purifying selection or even premature stop codons in the other *Leptopilina* genome. Interestingly, the phylogeny built on LbFVorf11 homologues suggests that the gene was acquired by *Filamentoviridae* from eukaryotes. This suggests a two-step integration process from eukaryotes to filamentoviruses and then to the genomes of wasps (see electronic supplementary material, figure S16-3).

A *lef-5* homologue was shared by all species (apart from *Leptolamina* sp.; electronic supplementary material, figure S16-13). Interestingly, in both LbFV and LhFV, *lef-5* has an alternative start codon , 'TTG'. This feature was conserved in the genomes of the wasps with the exception of *L. heterotoma* and *Trichoplasta* sp. that encode a classic 'ATG' start codon and of *Leptopilina clavipes* that encodes the alternative start codon , 'CTG'. The endogenized *lef-5* showed a complete ORF and d*N/*d*S* analysis suggests they are under strong purifying selection (mean d*N/*d*S* = 0.1118 (s.e. = 0.03)). This gene might be involved in the RNA polymerase initiation transcription factor, as in baculoviruses [36].

*LbFVorf44* EVE was found only in *Leptopilina boulardi*. It had multiple paralogues (*n* = 36) distributed in 23 scaffolds (electronic supplementary material, figure S16-4). One of the LbFVorf44 copies co-occurred in the same scaffold as the *lef-8* EVE. This finding suggests an integration of *LbFVorf44* along with the other genes during the same ancestral event, followed by several duplications in *L. boulardi* and subsequent loss in the other species. Alternatively, this may correspond to an independent *L. boulardi*-specific endogenization event with one paralogue fortuitously colocalized with *lef-8* EVE. A d*N/*d*S* analysis of these paralogues suggests that they evolve under purifying selection (mean d*N/*d*S* = 0.3004 (s.e. = 0.077)). This gene has no known homologues in public databases.

#### (ii) Second recent endogenization event unique to the *Rhoptromeris* sp. genome

The second recent event detected specifically in *Rhoptromeris* sp., involved 9 filamentous genes that ultimately led to the presence of 16 EVEs (this count is higher due to the presence of paralogues). Four of these genes were specific to *Rhoptromeris* sp., while five were detected in both the first and second events (figure 1). The phylogeny constructed on the concatenated EVEs positions *Rhoptromeris* sp. outside the Eucoilini clade, close to LbFV species, indicating acquisition from an independent event involving a virus related to LbFV, rather than LhFV. Among the EVEs, 7 out of 16 had premature stop codons or incomplete ORFs, suggesting non-functionality for some copies. However, the remaining nine EVEs may still be functional since they had complete ORFs. Interestingly, LbFVorf10 probably replaced the homologous EVE from the first event in *Rhoptromeris* sp. (figure 1*a*). Similarly to the first ancestral event, LbFVorf10 and LbFVorf11 were also found next to each other in the *Rhoptromeris* sp. genome, as they are in the genome of their closest relative LbFV, again suggesting that they entered jointly into wasp chromosomes. *Integrase* and LbFVorf106 (*Odv-e66*) were unique to *Rhoptromeris* sp. (figure 2*a*). No evidence of selective pressure was observed on the *Odv-e66* EVE (d*N/*d*S* not different from 1). The same d*N/*d*S* analysis was not feasible on the *Integrase* gene, due to the absence of paralogues.

### (c) All EVEs of the first ancestral endogenization event show signs of domestication

The d*N/*d*S* values obtained for the 18 EVEs acquired during the first ancestral event were low (mean = 0.18, s.d. = 0.084), and within the range of values observed for the highly conserved BUSCO genes (electronic supplementary material, figure S7). However, EVEs on average had slightly higher d*N/*d*S* values compared with BUSCO genes (two-sided *t*-test, d.f. = 1015, *p*‐value = 6.442 × 10^−09^), suggesting either a lower stabilizing selection intensity or diversifying selection in some sites. In total, 2354 codons (out of 7233) showed evidence of purifying selection, while 23 were likely evolving under diversifying selection, possibly indicating some interactions with host proteins (electronic supplementary material, figure S8).

### (d) The basal *Trybliographa rapae* produce VLPs, as *Leptopilina* species do

**Figure 3.**
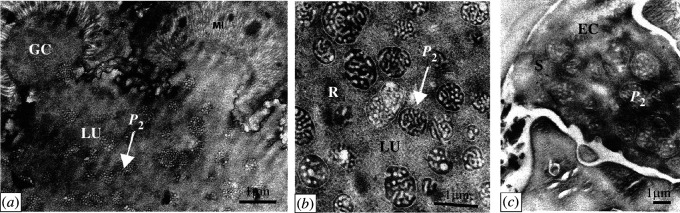
Virus-like particles in the venom gland of *Trybliographa rapae* as observed by transmission electron microscopy (Breton strain). (*a*) General view of the distal part of the venom gland. (*b*) Viral-like particles accumulate in the lumen of the venom gland reservoir. (*c*) Presence of VLPs in the egg canal of the ovipositor. EC, egg canal; GC, glandular cells; MI, microvilli; LU, gland lumen; *P*_2_, type 2 particles; S, secretions. The scale bars represent 1 µm in all three panels.

Electron microscopy investigations were performed on *Trybliographa rapae*, a close relative of the *Trybliographa* sp*.* used for the genomic analysis (<9% cytochrome c oxidase subunit 1 (CO1) divergence, see electronic supplementary material, figure S14). Two distinct types of VLPs either formed in the paired gland (particles called ‘P1’ hereafter, see electronic supplementary material, figure S11) or in the venom gland (also known as the unpaired gland, hereafter called ‘P2’, see figure 3*a*) were observed. P2 particles accumulate in the reservoir of the venom gland (figure 3*b*), similar to VLPs found in *Leptopilina* sp. Both types of particles were observed in the ovipositor canal, indicating that they are likely injected with the eggs during oviposition (figure 3*c*; electronic supplementary material, figure S11J). ‘Type 2’ particles (P2) share striking morphological similarities with VLPs produced by *Leptopilina* species (see particle comparisons in electronic supplementary material, figure S9). No evidence of host encapsulation was observed on several hundred laid eggs, suggesting that these VLPs (P1 and/or P2) confer immunosuppression to the wasp eggs. Interestingly, particles were abundant on the egg surface after oviposition in all three strains tested, although it was unclear whether they correspond to P1, P2 or both (electronic supplementary material, figure S11B,D–H). More information regarding VLP formation from the *Trybliographa rapae* venom gland can be found in electronic supplementary material, figure S10. In conclusion, the most basal species in the Eucoilini + Trichoplastini clade, namely *Trybliographa rapae*, produce, in their venom gland, VLPs having similar morphological and life-history characteristics as the ones observed in the more derived *Leptopilina* species.

### (e) Timing of the endogenization events

Previous analysis based on fossil calibration at the scale of the Cynipoidea estimated that the common ancestor of *Leptopilina* sp., *Trichoplasta* sp., *Rhoptromeris* sp. and *Trybliographa* sp. lived in the Cretaceous period, approximately 76 million years ago (55–100 Myr) [24]. Since our analysis shows that the endogenization event (event I) took place in this same common ancestor, we estimate that this major endogenization event occurred during this time period. Based on the same rationale, we estimated that the second endogenization event that only concerns *Rhoptromeris* sp. occurred in the last 40 million years since this is the estimated time period of the split between *Rhoptromeris* sp. and *Trichoplasta* sp. (confidence interval for this species node: 22–59 Myr).

## 3. Discussion

In this study, we analysed the diversity of cynipoid wasps observed to have a viral domestication event initially reported in *Leptopilina* [13]. From previous work, it was established that (i) VLPs protect eggs from encapsulation in *Leptopilina* species [16], and that (ii) in *Leptopilina boulardi*, for which various experimentations and genomic analysis have been conducted, this VLP production was linked to the presence of 13 virally derived genes [13]. Because all *Leptopilina* species analysed so far produce VLPs [13] and because all *Leptopilina* genomes analysed so far contain the same conserved LbFV-like genes, it was concluded that all *Leptopilina* species rely on those viral genes to produce the VLPs [13]. Here, after analysing wasp genomes from five Figitidae subfamilies, we propose to extend this reasoning to the whole clade of Eucoilini + Trichoplastini. Indeed, in this clade, all species share a set of 15 highly conserved viral genes (including the 13 previously identified by [13]), and most importantly, the most basal species within this clade, namely *Trybliographa rapae* produce VLPs. This finding thus suggests that VLP production is a common feature shared by all species of this clade, not only *Leptopilina* species, supporting the hypothesis that these virally derived genes are responsible for VLP production.

Building on the recent sequencing of additional LbFV-related viruses [7], this analysis also provided a comprehensive view of the set of viral genes involved in this event. Five additional viral genes endogenized during the same event were identified, including a *Naldaviricetes* core gene involved in transcription (*lef-5*). The overall picture is that all the core genes involved in transcription, as well as some essential genes involved in DNA replication (including the viral DNA polymerase), have been retained by selection in all wasp genomes of this clade. This conservation strongly suggests that this machinery is essential for wasp fitness and is likely involved in genomic amplification and associated transcription of virally derived genes, as observed in the venom gland of developing *L. boulardi* females [13]. Additionally, the *Ac81* gene that plays a crucial role in baculovirus envelopment [37] is shared by all wasp genomes. This suggests that it plays a critical role in the production of the VLP envelope, as also attested by the presence of its proteinic product in mature particles purified from *Leptopilina* species [13]. Both features (transcriptomic and amplification machinery on one side and the presence of *Ac81*) are shared with other VLP systems previously described in the distantly related Ichneumonoidea *Venturia canescens* [11] and *Fopius arisanus* [12]. On the contrary, the picture is completely different on *per os* infectivity factors (PIFs), which are major players for the entry of baculovirus into insect cells. While the genomes of both the *Venturia* and *Fopius* systems encode most of the known PIFs, none are encoded by the Eucoilini + Trichoplastini genomes. Knowing that VLPs from *Leptopilina* permit the delivery of virulence proteins into *Drosophila* immune cells [38], indicates that other mechanisms not relying on PIF proteins have been recruited in Eucoilini + Trichoplastini.

The best understood case of viral domestication in parasitoids involves Braconidae belonging to the microgastroid complex. In this system, the (braco-)virus was acquired by the ancestral wasps around 103 Myr from a nudivirus ancestor [9,39]. Among the subfamilies that compose this clade, the Microgastrinae is by far the most speciose and rapidly radiated around 50 Myr [39]. It has been hypothesized that this rapid radiation is correlated with a rapid radiation in their lepidopteran hosts, which are also very diverse [40]. One hypothesis that was put forward to explain the diversification of this particular subfamily compared with Cheloninae, is that they develop from inside Lepidoptera larvae (while Cheloninae develop from within the eggs) and as such are exposed to the host immune system, since eggs are much less active immunologically than larvae. The idea is that endoparasitoids attacking larvae require specific adaptations for each host species attacked, thus favouring the ecological speciation process.

Similar to microgastroids, we found that ancestral filamentovirus domestication also affected a diverse, albeit smaller, clade of wasps (the Eucoilini and Trichoplastini tribes) that diversified around 75 Myr. All these species are koinobiont endoparasitoids that attack the larvae of distinct species of Schizophora Diptera in various environments [41]. For instance, *Leptopilina* develops from Drosophilidae, *Rhoptromeris* from Chloropidae flies, *Trybliographa* from Anthomyiidae flies and *Trichoplasta* develops from Drosophilidae, Muscidae and Lonchaeidae flies [41]. It is interesting to note that the Schizophora clade, which is the most species-rich group in Cyclorrhapha [26], underwent rapid radiation just after the Cretaceous–Palaeogene (K–Pg) crisis, starting around 65 to 68 million years ago depending on the estimates [42,43] (electronic supplementary material, figure S12). The domestication of the virus in Eucoilini + Trichoplastini thus coincides quite well with this period [41]. We can speculate that the acquisition of the virus favoured adaptation to these various hosts, allowing the wasps to cope with the specificities of each host immune system.

The lack of evidence for filamentovirus domestication in *Ganaspis* is remarkable. Members of this genus tend to parasitize the same hosts as *Leptopilina*, often in direct competition with them [44–46]. In some cases, it appears *Ganaspis* can actually push *Leptopilina* from a given niche space [45], but in other cases, *Leptopilina* can keep *Ganaspis* at bay [47]. As such, we predict that another form of host immune system manipulation is at play within *Ganaspis*, and is certainly worthy of additional study.

Finally, our analysis also provided evidence for a second event of endogenization involving a closely related donor virus, also belonging to the newly proposed *Filamentoviridae* family [7]. While we estimate that the major endogenization event occurred approximately 75 million years ago, soon after the separation of the Eucoilini + Trichoplastini lineage, the more recent endogenization took place in the last 40 million years, within the branch leading to the genus *Rhoptromeris*. It is unclear whether this second event provided a selective advantage to the wasps since d*N/*d*S* analysis was only possible for a few genes having a few paralogues and did not provide evidence for purifying selection. However, it is still possible that some genes acquired during the ancestral endogenization event that were subsequently lost in this clade were replaced by genes from this second endogenization event.

In conclusion, this study shows that the newly proposed viral family *Filamentoviridae* [7], which is involved in both events, has a long and intimate history of association with endoparasitoid wasps. More generally, this work combined with previous literature [8–12] shows that DNA viruses associated with parasitoid wasps had a strong impact on the evolution and diversification of the parasitoid lifestyle in very distant wasp clades.

## 4. Material and methods

### (a) *Cynipoidea* specimens and DNA extractions

DNA extractions were performed using the NucleoSpin Tissue Macherey kit following the manufacturer's instructions. Either metasoma (10/41) or whole body (31/41) were used and the elution was done in 20 µl of Tris-EDTA buffer. See electronic supplementary material, table S2 for voucher information.

### (b) PCR amplification of ORF96

Using the primers described in [13] (40 cycles of: 95°C for 30 min, 57.8°C for 30 min, 72°C for 60 min) 411 bp of the most conserved viral gene LbFV_ORF96 were PCR amplified. The CO1 marker was also amplified in 33 out of 41 specimens, indicating that the DNA extraction was successful (using the following PCR settings (40 cycles of: 95°C for 30 min, 48°C for 30 min, 72°C for 60 min)) for these specimens. Except for two species, two specimens were analysed this way.

### (c) Genome assembly and quality check

The DNA of single females was used to construct TruSeq Nano DNA (350) Illumina libraries. The 30 Gb of paired-end reads (2 × 150 bp) obtained for each sample were cleared from duplicates using SuperDeduper v. 1.3.0 [48] (-fnodup), quality trimmed using Fastp v. 0.22.0 (–cut_tail –length_required 100 –correction) and assembled using Megahit v. 1.2.9 [49] (–kmin-1pass), followed by a scaffolding step using SOAPdenovo-fusion (-D-s) and reduction using the Redundans pipeline [50] (default parameters). The quality check was done using the BUSCO pipeline v. 5.3.0 [51] on the Hymenoptera and Arthropoda databases and assembly statistics were computed using Quast [52] (electronic supplementary material, table S1).

### (d) Homology with *Filamentoviridae*

All Cynipoidea genomes were screened for the presence of *Filamentoviridae*-like ORFs with the Mmseqs2 search algorithm [53] (e-value max = 10^-3^ and option -s 7) using the predicted proteins from the *Naldaviricetes* as queries (see details in [7]). EVE candidates were then clustered following the pipeline used in [7].

### (e) Arguments for endogenization

To ensure candidate EVEs are really encoded in the wasp genomes (and not exogenous), we looked for the presence of insect genes along the scaffolds, assuming that the presence of several insect genes in a viral scaffold is unlikely. Gene prediction was made using the Metaeuk easy-predict workflow [54] (default parameters on the UniProtKB database) and taxonomic assignment was done using the taxtocontig workflow. For each contig, we adopted a majority voting strategy among the taxonomically labelled proteins to assign taxonomy to the contig.

The presence of transposable elements in a contig was also considered a marker of its eukaryotic nature since very few viral genomes contain transportable elements [55–59], likely due to the fact that TEs are highly deleterious and quickly eliminated by selection in viral populations [57,58]. We searched for transposable elements using a BlastX-like approach (implemented in Mmseqs2 search-s 7.5), taking as query the scaffolds of interest and as database the TEs of the RepeatModeler database (RepeatPeps, v. 2.0.2, [60])(e-value < 1 × 10^−10^ alignment length > 100 aa). Overlapping hits were merged. The coverage and GC content of the candidate scaffolds were also measured and compared with BUSCO-containing scaffolds (electronic supplementary material, figure S4).

### (f) EVE phylogenies and event assignations

Proteins were aligned using ClustalO (v. 1.2.4) [61] and the phylogenies were inferred using Iqtree (v. 2.1.2) [62] (options -m MFP-alrt 5000-B 5000). The phylogenetic tree was then used to infer endogenization events. Shared events are expected to be represented by a monophyletic clade of related wasp species nested within a clade of filamentoviruses. For each set of homologous sequences, we thus grouped wasp sequences into a single event, if they formed a well-supported monophyletic clade (sequences) (with a bootstrap score > 80).

By doing so, we noticed that three topologies could be observed (electronic supplementary material, figure S5), corresponding to different scenarios (see §2). This information was used to assign the sequences to the two major events (so-called ‘ancient’ in §2 when shared by all Eucoilini+Trichoplastini and ‘recent’ when concerning only *Rhoptromeris* sp.).

Additionally, we used physical proximity to further refine the assignment of EVEs to the same endogenization event. If multiple (non-homologous) EVEs were found within the same scaffold in a given species, they were assigned to the same endogenization event, based on the rationale that this colocalization is very unlikely in the case of independent endogenization events. In case the different homologous sequences were also detected in another species, they were also aggregated into the same event, even in the absence of scaffold colocalization in that second genome assembly. By applying this principle in a transitive way (if A and B colocalize in species 1, and B and C colocalize in species 2, then A, B and C are likely derived from the same ancestral event) most EVEs were aggregated into the two main events.

### (g) Rearing protocol and microscopic analysis of *T. rapae* strains and eggs

Most microscopic observations were performed using the Breton strain of *T. rapae* that was reared on *Delia radicum* larvae infesting Cruciferae roots using the technique described by [63]. Additionally, two strains from Quebec and Finland were used to check the presence of particles on the egg surface after oviposition (electronic supplementary material, figure S11B,D–H). Second-stage host larvae were subjected to parasitism by females for 5 min, allowing to determine the precise time between egg laying and recovery. Ovarian eggs were obtained by dissection of the ovary, and laid eggs by dissection of the parasitized larvae in Ringer’s physiological solution. For scanning electron microscopy, samples were fixed with 2.5% glutaraldehyde buffered with sodium cacodylate for 1 h, dehydrated for 10 min in baths of 70°, 80°, 90°, 95° and twice 100° alcohol, followed by 100° acetone. They were then dried using the critical point drying technique (CPD), liquid dried on a Balzers CPD10, metallized with palladium gold using a cathodic sputter and observed on a JEOL JSM 6400. Samples observed by transmission electron microscopy were fixed with cacodylate-buffered gluraldehyde 2.5, placed in a 2% buffered osmium tetroxide solution for 1 h and dehydrated with pure acetone. They were embedded in Epon-Araldite. Semi-fine sections were taken on a Reichert OM.U2 ultramicrotome. Sections were stained with 1% toluidine blue and observed using Phillips CM-12 transmission electron microscopy. For full details, see [64].

## Data Availability

All sequencing data are available under the NCBI BioProject PRJNA831620. All pipelines, statistical as well as figure scripts are available under the Dryad repository [65]. Supplementary material is available online [66].
